# Environmental Footprint of a Low-Fat Vegan Diet in People With Type 1 Diabetes: A Secondary Analysis of a Randomized Clinical Trial

**DOI:** 10.1016/j.cdnut.2026.107709

**Published:** 2026-04-30

**Authors:** Reagan Smith, Arathi Jayaraman, Brighid McKay, Laura Chiavaroli, Songhee Back, Ilana Fischer, Richard Holubkov, Neal D Barnard, Hana Kahleova

**Affiliations:** 1 Physicians Committee for Responsible Medicine, Washington, DC, United States; 2 Department of Nutritional Sciences, Temerty Faculty of Medicine, University of Toronto, Canada; 3 Toronto 3D Knowledge Synthesis and Clinical Trials Unit, Clinical Nutrition and Risk Factor Modification Centre, St. Michael’s Hospital, Toronto, Ontario, Canada; 4 Li Ka Shing Knowledge Institute, St. Michael’s Hospital, Toronto, Ontario, Canada; 5 School of Medicine, University of Utah, Salt Lake City, Utah, United States; 6 Adjunct faculty, George Washington University School of Medicine and Health Sciences, Washington, DC, United States

**Keywords:** environmental footprint, cumulative energy demand, greenhouse gas emissions, diet, plant-based, vegan, nutrition

## Abstract

A 12-wk randomized clinical trial found that adults with type 1 diabetes on a low-fat, vegan diet (*n* = 29) showed greater improvements in insulin sensitivity compared with a portion-controlled diet (*n* = 29). This secondary analysis calculates the greenhouse gas emissions (GHGEs) and cumulative energy demand (CED) of both diets by linking 3-d dietary records to external data sources. A repeated-measures analysis of variance was performed unadjusted and adjusted for change in energy intake. GHGE decreased in the vegan group by 55% (*P* = 0.001), compared with no change in the portion-controlled group (between-group *P* = 0.01). CED decreased in the vegan group by 44% (*P* = 0.01), with no change in the portion-controlled group (between-group *P* = 0.22). Energy intake was not a significant predictor of the observed changes. The decrease in GHGE and CED in the vegan group likely reflects reduced meat and dairy intake, suggesting that prioritizing plant-based foods over animal products may effectively reduce dietary environmental impact.

This trial was registered at clinicaltrials.gov as NCT04944316 (https://clinicaltrials.gov/study/NCT04944316).

## Introduction

Dietary choices are consequential to both human health and the environment, with food systems accounting for an estimated one-third of global greenhouse gas emissions (GHGEs) [1]. As previously reported [2], a low-fat vegan diet improved insulin sensitivity and glycemic control compared with a portion-controlled diet in adults with type 1 diabetes (T1D). This secondary analysis assessed the environmental impact, specifically GHGE and cumulative energy demand (CED), of a low-fat vegan diet. Although individuals with T1D often follow dietary strategies focused on carbohydrate management, the environmental impact of dietary patterns is primarily determined by food composition and is therefore broadly applicable to the general population.

## Methods

Previously described in detail [2], the 12-wk study took place in Washington, DC, between August 2021 and November 2022, as a single-center, 1:1 randomized, open parallel trial. The enrolled 58 adult men and women had diagnoses of T1D and reported stable insulin regimens for ≥3 mo prior. Those with type 2 or gestational diabetes, BMI of ≥40 kg/m^2^, Hemoglobin A1c (HbA1c) of ≥12%, pregnancy or lactation, tobacco use, alcohol or substance use disorder, or evidence of an eating disorder were excluded. In accordance with study protocol, approved in February 2021 by the Chesapeake Institutional Review Board (protocol number Pro00048903), all participants provided written informed consent.

Participants were randomly assigned to a low-fat vegan diet (*n* = 29) or a portion-controlled diet (*n* = 29). Those on the low-fat vegan diet were instructed to favor low glycemic index foods and avoid animal products and added fats, for a daily fat intake limited to ∼30 g. The diet consisted of fruits, vegetables, legumes, and whole grains without calorie or carbohydrate restrictions. The portion-controlled diet kept carbohydrate intake stable while reducing daily energy intake by 500 to 1000 kcal for overweight participants. Both groups received online nutrition education classes and support from registered dieticians weekly and recorded the content of all meals using the Cronometer nutrition tracking application.

Study participants received guidance to maintain their existing exercise habits and continue their pre-existing medication regimens for the duration of the study, except as modified by their personal physicians; insulin doses were adjusted in response to repeated hypoglycemic events. All outcomes were assessed at baseline and 12 wk. The basal and bolus insulin units injected per day were averaged from 3 consecutively tracked days. Participants also completed 3-d dietary records, which were analyzed by a registered dietician certified in the Nutrition Data System for Research [3].

For the secondary analysis, intakes from dietary records were linked to the United States Food Commodity Intake Database [4] and database of Food Impacts on the Environment for Linking to Diets [5] by independent reviewers blinded to group assignment to calculate GHGE and CED. A senior researcher blinded to group assignment verified linking accuracy and a statistician blinded to dietary interventions used a repeated-measure analysis of variance, with all results presented as means with 95% confidence intervals (CIs). Additional analyses were performed adjusting for change in total energy intake as a covariate.

## Results

### Characteristics of the participants

Of the 377 people who completed an initial screening, 58 met participation criteria and were randomly assigned to the vegan (*n* = 29) or control (*n* = 29) groups. Dietary intake, food acceptability, physical activity, and anthropometric and laboratory variables, as well as the continuous glucose monitoring data in response to 12 wk of each diet, have been reported previously [2].

### Environmental footprint

**FIGURE 1:**
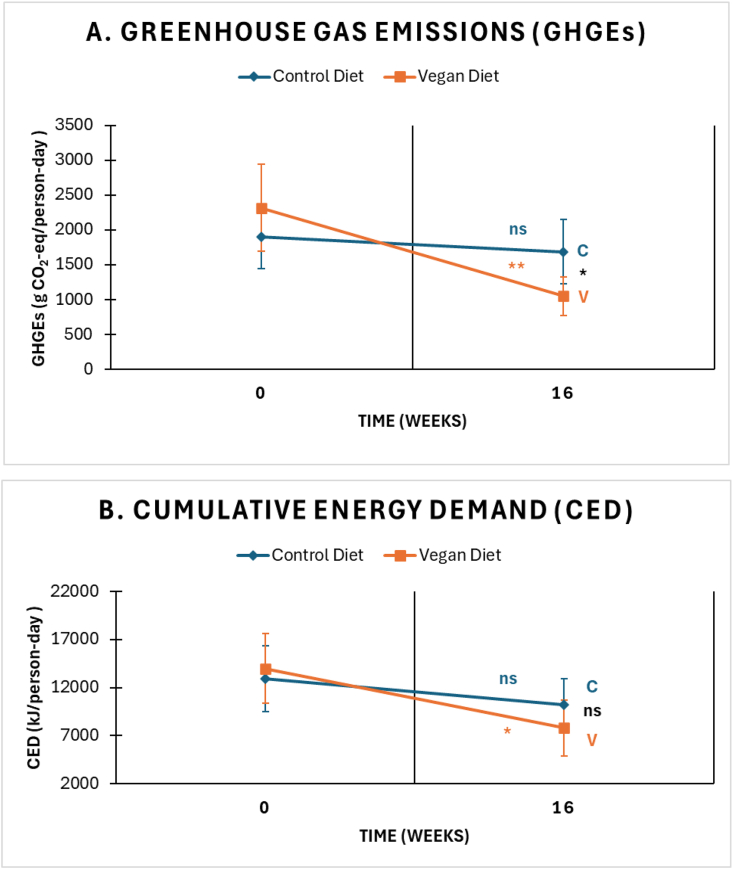
Changes in total greenhouse gas emissions (A) and cumulative energy demand (B) in the control (C) and vegan (V) groups from baseline to week 12. The data are shown as means with 95% confidence intervals. ∗*P* < 0.05; ∗∗*P* < 0.01; ∗∗∗*P* < 0.001; ns, nonsignificant.

GHGE decreased (*P* = 0.001) in the vegan group by 55% (−1267 g CO_2_-eq/person-day), with no change in the portion-controlled group (effect size: −1054 g CO_2_-eq/person-day; 95% CI: −1888, −220; *P* = 0.01) (Figure 1, Table 1). The decrease in the vegan group was mainly attributable to the reduced consumption of meat (effect size: −1189 g CO_2_-eq/person-day; 95% CI: −1879, −500; *P* = 0.001). CED decreased (*P* = 0.01) in the vegan group by 44% (−6196 kJ/person-day), whereas there was no change in the portion-controlled group; the between-group difference was not significant (effect size: −3520 kJ/person-day; 95% CI: −9203, 2163; *P* = 0.22) (Table 1). Again, most of the reduction was attributable to reduced meat intake (effect size: −4890 kJ/person-day; 95% CI: −8677, −1103; *P* = 0.01), followed by reduced dairy consumption (effect size: −1324 kJ/person-day; 95% CI: −2431, −216; *P* = 0.02). Adjustment for change in energy intake did not materially alter the observed reductions in GHGE and CED in the vegan group. Energy intake was not a significant predictor of the observed changes (*P* = 0.23 for GHGE and *P* = 0.06 for CED).

**TABLE 1:** Changes in greenhouse gas emissions (GHGEs) and cumulative energy demand (CED) from specific food groups at baseline and week 12 in the control and vegan groups, ranked according to the magnitude of their impact on changes in the metrics.

	Control group			Vegan group			Effect size	*P* value
	Week 0	Week 12	Change	Week 0	Week 12	Change		
Total GHGE (g CO_2_eq/person-day)	1899.8 (1449.1, 2350.5)	1686.8 (1226.8, 2146.8)	−213 (−716.1, +290)	2317.7 (1698.3, 2937.1)	1050.9 (773.1, 1328.6)	−1266.9 (−1961.1, −572.6)∗∗	−1053.8 (−1887.6, −220.1)	0.0148
Meat	436.8 (164.4, 709.2)	495.3 (180.7, 809.8)	58.5 (−262.6, +379.6)	1130.8 (500.6, 1761)	0 (0, 0)	−1130.8 (−1761, −500.6)∗∗	−1189.3 (−1878.9, −499.7)	0.0015
Dairy	305.6 (131.5, 479.7)	252.9 (96.8, 408.9)	−52.7 (−244.7, +139.3)	293 (193.7, 392)	0 (0, 0)	−292.9 (−392, −193.7)∗∗∗	−240.1 (−450.7, −29.6)	0.0271
Added sugar	60.2 (30.6, 89.8)	72 (43.7, 100.3)	11.8 (−14.3, +37.8)	46.5 (20.3, 72.7)	18.6 (5.3, 31.9)	−28 (−57.5, +1.5)	−39.7 (−77.8, −1.7)	0.0412
Added fats	79.3 (26.5, 132)	44.4 (26.9, 61.9)	−34.9 (−82.5, +12.7)	46.3 (27, 65.6)	14.9 (6.4, 23.3)	−31.4 (−53.3, −9.6)∗∗	3.4 (−47.8, +54.6)	0.8906
Vegetables	161.6 (110.3, 213)	211.7 (145.3, 278)	50 (−36.6, +136.7)	208.7 (135.1, 282.4)	262.3 (182.6, 342)	53.6 (−10.2, +117.4)	3.6 (−99, +106.2)	0.9440
Grains	156.7 (125.9, 187.5)	152.5 (95.3, 209.7)	−4.2 (−56.4, +47.9)	171.9 (120.1, 223.6)	191.6 (144.5, 238.7)	19.8 (−42.1, +81.6)	24 (−54.3, +102.3)	0.5373
Eggs	155.4 (67.6, 243.3)	43.1 (3.7, 82.5)	−112.3 (−215.4, −9.2)∗	96.2 (46.1, 146.3)	14.3 (−15.8, 44.4)	−81.9 (−144.7, −19.2)∗	30.4 (−84.1, +144.9)	0.5924
Nuts and seeds	108.4 (3.4, 213.5)	58.1 (25.2, 91)	−50.4 (−145.3, +44.6)	24.6 (2.4, 46.9)	12.4 (1.2, 23.7)	−12.2 (−38.2, +13.8)	38.2 (−59.3, +135.6)	0.4218
Legumes	32.1 (14.7, 49.6)	50.5 (16.4, 84.5)	18.3 (−18.1, +54.8)	11.7 (3.1, 20.4)	91.5 (67.2, 115.9)	79.8 (+54.1, +105.5)∗∗∗	61.5 (+19, +104)	0.0059
Fruit	114.1 (53.7, 174.5)	127.3 (72.4, 182.2)	13.2 (−50.7, +77)	90.1 (51.4, 128.7)	184.5 (105.8, 263.3)	94.5 (+20.1, +168.9)∗	81.3 (−13.5, +176.2)	0.0905
Total CED (kJ/person-day)	12,902.7 (9476.7, 16,328.7)	10,227.1 (7486.1, 12,968.1)	−2675.6 (−6230.1, +879)	13,996.1 (10,361.5, 17,630.7)	7800.4 (4900.1, 10,700.7)	−6195.7 (−10,845.9, −1545.5)∗	−3520.1 (−9203.3, +2163.1)	0.2164
Meat	2888.8 (1223.8, 4553.8)	2048.9 (941.4, 3156.5)	−839.9 (−1956.5, +276.7)	5730.2 (2063.6, 9396.9)	0 (0, 0)	−5730.2 (−9396.9, −2063.6)∗∗	−4890.4 (−8677.4, −1103.4)	0.0140
Dairy	1602.6 (707.5, 2497.7)	1341.1 (514.7, 2167.5)	−261.5 (−1264.8, +741.8)	1585 (1047.8, 2122.2)	0 (0, 0)	−1585 (−2122.2, −1047.8)∗∗∗	−1323.5 (−2431.2, −215.9)	0.0211
Added fats	511.9 (111.5, 912.2)	525.2 (−57.4, 1107.9)	13.4 (−574.3, +601)	484.2 (202, 766.4)	0 (0, 0)	−484.2 (−766.4, −202)∗∗	−497.5 (−1134.1, +139)	0.1195
Added sugar	456.3 (240.4, 672.1)	566.9 (339.7, 794.1)	110.6 (−86, +307.2)	346.7 (162.1, 531.4)	155.7 (41.6, 269.7)	−191.1 (−405.7, +23.6)	−301.7 (−582.8, −20.6)	0.0362
Vegetables	1087.5 (748, 1427.1)	1415.4 (1002.5, 1828.4)	327.9 (−225.8, +881.7)	1499.4 (942.8, 2056)	1811.6 (1285.7, 2337.4)	312.1 (−194.8, +819)	−15.8 (−736.7, +705.1)	0.9647
Grains	959.9 (758.5, 1161.4)	949.7 (591.9, 1307.5)	−10.2 (−343, +322.5)	1013.4 (720.9, 1305.8)	1143.3 (868.6, 1417.9)	129.9 (−208, +467.8)	140.1 (−316.8, +597)	0.5369
Nuts and seeds	543.4 (22.5, 1064.2)	306.2 (139.3, 473.1)	−237.2 (−709.9, +235.6)	126.9 (14.2, 239.6)	62.7 (6.7, 118.7)	−64.2 (−195.9, +67.5)	173 (−312.6, +658.6)	0.4645
Eggs	935.9 (407, 1464.8)	259.6 (22.5, 496.6)	−676.3 (−1296.9, −55.7)∗	579.1 (277.3, 880.9)	86 (−95.4, 267.4)	−493.1 (−870.9, −115.3)∗	183.2 (−506.2, +872.6)	0.5924
Legumes	161.5 (73.6, 249.3)	253.6 (82.7, 424.5)	92.1 (−91, +275.2)	58.8 (15.3, 102.3)	459.8 (337.5, 582.1)	401 (+271.9, +530.1)∗∗∗	308.9 (+95.5, +522.3)	0.0059
Fruit	928.3 (423, 1433.7)	1080.8 (593.5, 1568)	152.4 (−368.4, +673.2)	774.8 (461.5, 1088)	1511.6 (891.1, 2132.1)	736.9 (+153.8, +1319.9)∗	584.4 (−171.1, +1340)	0.1251

The results are presented as mean daily intakes with 95% confidence intervals: ∗*P* < 0.05; ∗∗*P* < 0.01; and ∗∗∗*P* < 0.001.

## Discussion

This secondary analysis of the 12-wk randomized clinical trial showed a 55% reduction in GHGE and a 44% reduction in CED on a low-fat vegan diet, with no significant changes on the portion-controlled diet. The reduction in GHGE is approximately equivalent to the emissions associated with driving car for ∼3 miles/d, and the reduction in CED is approximately equivalent to the energy needed to run a household refrigerator for ∼1 to 2 d [6]. These findings add to the body of evidence that links dietary patterns favoring plant products to lower GHGE than those high in meat and dairy consumption [7]. Energy intake was not a significant predictor of changes in GHGE or CED, further supporting that dietary composition, rather than caloric intake, was the primary driver of environmental impact.

The strengths of the current study include a randomized, parallel design. Although this study was conducted in individuals with T1D, the environmental impact observed is driven by dietary composition and is likely generalizable to broader populations adopting similar dietary patterns. Importantly, the primary trial also demonstrated improved food acceptability in the vegan group, supporting the feasibility and sustainability of this dietary pattern. The study also has limitations. Food consumption was based on self-reported diet records. The between-group difference in CED did not reach statistical significance and should be interpreted with caution. The participants were research volunteers and may not represent the general population.

In conclusion, in this randomized study in adults with T1D, a low-fat vegan diet was associated with significant within-group reductions in GHGE and CED, important drivers of climate change.

## Author contributions

The authors’ responsibilities were as follws—HK, NDB: designed and executed the study; AJ, BM, SB: linked the dietary intakes with the United States Department of Agriculture Food Commodity Intake Database and the database of Food Impacts on the Environment for Linking to Diets; LC: reviewed the accuracy of the links; IL, RS: prepared the data for analysis; RH: performed the statistical analysis; and all authors: had full access to the data, contributed to the manuscript, and approved its final version. HK had full access to all the data in the study and takes responsibility for the integrity of the data and the accuracy of the data analysis.

## Data availability

Data described in the manuscript will be made available upon request, pending application and approval.

## Declaration of Generative AI and AI-assisted technologies in the writing process

The authors declare that no generative AI or AI-assisted technologies were used in the writing of this manuscript.

## Funding

This work was funded by the Physicians Committee for Responsible Medicine.

## Conflict of interest

HK, AJ, IF, RS, BM, SB, and HK received compensation from the Physicians Committee for Responsible Medicine for their work on this study. LC has received research funding from the Canadian Institutes of Health Research (CIHR), Protein Industries Canada (a Government of Canada Global Innovation Cluster), the United Soybean Board (The United States Department of Agriculture Soybean “Check-off” Program), and the Alberta Pulse Growers. She has received honoraria from the Arkansas Children’s Hospital and Plant-Based Health Professionals UK. NDB is an Adjunct Professor of Medicine at the George Washington University School of Medicine.
